# Structure of a *Talaromyces pinophilus* GH62 arabinofuranosidase in complex with AraDNJ at 1.25 Å resolution

**DOI:** 10.1107/S2053230X18000250

**Published:** 2018-07-26

**Authors:** Olga V. Moroz, Lukasz F. Sobala, Elena Blagova, Travis Coyle, Wei Peng, Kristian B. R. Mørkeberg Krogh, Keith A. Stubbs, Keith S. Wilson, Gideon J. Davies

**Affiliations:** a York Structural Biology Laboratory, Department of Chemistry, The University of York, York YO10 5DD, England; b School of Molecular Sciences, The University of Western Australia (M313), 35 Stirling Highway, Crawley, WA 6009, Australia; c Fungal Diversity, Novozymes A/S, China Headquarters, 14 Xinxi Road, Shangdi Zone, Haidian District, Beijing 100085, People’s Republic of China; d Protein Biochemistry and Stability, Novozymes A/S, Krogshøjvej 36, 2880 Bagsværd, Denmark

**Keywords:** biofuels, glycosidases, enzymes, enzyme inhibitors, *Talaromyces pinophilus*, arabinofuranosidase

## Abstract

The enzymatic hydrolysis of complex plant biomass is a major societal goal of the 21st century in order to deliver renewable energy from nonpetroleum and nonfood sources. One of the major problems in many industrial processes, including the production of second-generation biofuels from lignocellulose, is the presence of ‘hemicelluloses’ such as xylans which block access to the cellulosic biomass. Xylans, with a polymeric β-1,4-xylose backbone, are frequently decorated with acetyl, glucuronyl and arabinofuranosyl ‘side-chain’ substituents, all of which need to be removed for complete degradation of the xylan. As such, there is interest in side-chain-cleaving enzymes and their action on polymeric substrates. Here, the 1.25 Å resolution structure of the *Talaromyces pinophilus* arabinofuranosidase in complex with the inhibitor AraDNJ, which binds with a *K*_d_ of 24 ± 0.4 µ*M*, is reported. Positively charged iminosugars are generally considered to be potent inhibitors of retaining glycosidases by virtue of their ability to interact with both acid/base and nucleophilic carboxylates. Here, AraDNJ shows good inhibition of an inverting enzyme, allowing further insight into the structural basis for arabinoxylan recognition and degradation.

## Abstract 2

The three-dimensional structure of a fungal arabinofuranosidase from CAZY family GH62 has been solved at 1.25 Å resolution in complex with the bespoke arabinofuranosidase inhibitor AraDNJ, shedding light on the activity of this catalyst in the enzymatic deconstruction of arabinoxylans.

## 1. Introduction   

**Figure 1:**
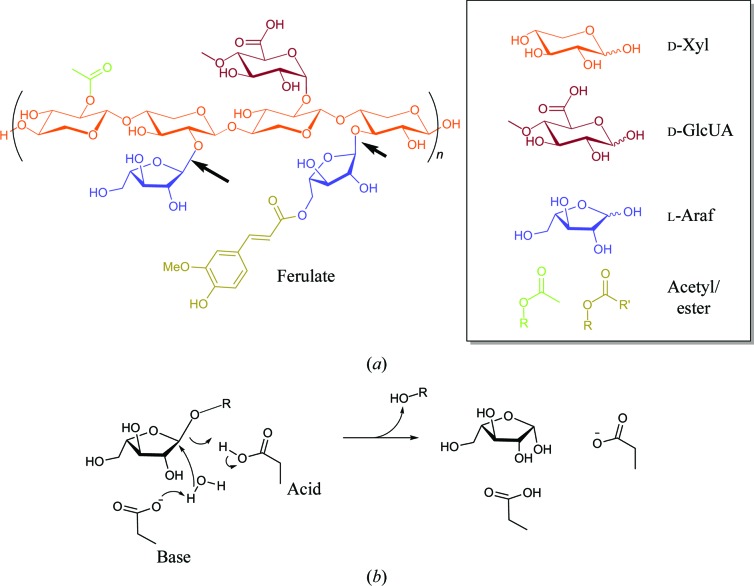
Xylans and their degradation. (*a*) The structure of a generic xylan, colour-coded by chemical group. Arrows indicate the positions of cleavage by arabinoxylan-active arabinofuranosidases. (*b*) The reaction scheme for an inverting arabinofuranosidase, which requires the presence of both Brønsted acid and base residues.

The production of ‘second-generation’ biofuels, *i.e.* from nonfood plants, is a major societal goal as we move away from petroleum-based energy towards secure and renewable energy. Although the majority of polysaccharide biomass in plants is cellulose, the cellulose fibres are coated with hemicelluloses such as xylan, which render access to the cellulose more difficult. Enzymatic degradation of xylan is therefore necessary for the action of cellulase on higher plants, but it is also an important substrate in itself in that glucose and xylan, with small quantities of other sugars, are the major substrates for biofuel generation (discussed in Somerville, 2007 ▸). The enzymatic degradation of hemicelluloses such as xylan is of major importance in the biofuel industry (reviewed in Pauly & Keegstra, 2008 ▸) and also in diverse industries such as bread manufacture, animal feed and the pulp and paper industry (for pulp bleaching). Xylan, which is a major component of the plant cell wall, consists of a backbone β-1,4-linked d-xylosyl chain, which is decorated with diverse substituents including 2- and 3-linked arabinofuranosyl moieties (typically in cereal arabinoxylans) and glucuronic acid (notably in cereal and hardwood glucuronoxylans). Xylan complexity is further segmented through ester-linked species such as acetyl and ferulate species, with the latter potentially linking the xylan to lignin (Fig. 1 ▸ *a*). Degradation of xylan both in natural environ­ments and in the industrial milieu therefore requires a plethora of enzymes, with some of the main players including β-xylanases, β-xylosidases, α-glucuronidases, acetyl and ferulate esterases and arabinofuranosidases, all of which are subject to keen academic and industrial study (recently comprehensively reviewed by Biely *et al.*, 2016 ▸).

Arabinoxylans, by virtue of being found in many of the plants now favoured for biofuel production, are considered to be a major ‘feedstock’ if we are to attain these societal goals in terms of renewable and secure energy (for reviews, see, for example, Lagaert *et al.*, 2014 ▸; Pauly & Keegstra, 2008 ▸). Given that arabinoxylan degradation requires a consortium of enzymes acting in partial synergy, most elegantly emphasized through Gilbert’s recent work on xylan degradation by the microbiota (Rogowski *et al.*, 2015 ▸), there is much interest in the structure, mechanism and specificity of xylan-active enzymes, with a special focus on side-chain-cleaving enzymes and their potential synergy with backbone-cleaving xylanases. This potential synergy is further complicated by the differing capacities of the endoxylanases themselves to accommodate side chains. Of particular interest are the arabino­furanosidases, which are capable of removing the arabinofuranosyl (Araf) substituents from the 2- and 3-positions of the xylan backbone, thus opening up the xylan backbone for attack by classical endoxylanases. Arabinofuranosidases are found in families GH2, GH3, GH43, GH51, GH54 and GH62 of the CAZy sequence-based classification (http://www.cazy.org; Lombard *et al.*, 2014 ▸).

CAZY family GH62 contains many enzymes that act as arabinoxylan-active arabinofuranosidases (extensively reviewed in Wilkens *et al.*, 2017 ▸). The first three-dimensional structures of GH62 enzymes appeared in 2014, with structures reported from the bacteria *Streptomyces coelicolor* (Maehara *et al.*, 2014 ▸) and *S. thermoviolaceus* (Wang *et al.*, 2014 ▸) and of two fungal enzymes from *Ustilago maydis* and *Podospora anserina* (Siguier *et al.*, 2014 ▸). The three-dimensional structures share a common five-bladed β-propeller fold with an active centre consistent with hydrolysis with inversion of anomeric configuration, with conserved Glu and Asp residues acting as the catalytic acid and catalytic base, respectively, in the single-displacement mechanism (Fig. 1 ▸ *b*). GH62 enzymes have been reviewed in CAZYpedia (for a review, see The CAZypedia Consortium, 2018 ▸).

Here, we present the three-dimensional structure of a fungal GH62 arabinofuranosidase from *Talaromyces pinophilus* refined at 1.25 Å resolution in complex with the bespoke iminosugar arabinofuranosidase inhibitor 1,4-dideoxy-l,4-imino-l-arabinitol (AraDNJ). The complex sheds light on the active site and, in light of previously published data, allows analysis of how the enzyme interacts with arabino­xylan substrates, serving to remove these side chains from the xylan backbone.

## 2. Materials and methods   

### 2.1. Macromolecule production and small-molecule synthesis   

The enzyme (a single-module GH62 arabinofuranosidase with no predicted N-glycosylation sites; GenBank MG656406) was cloned and expressed by standard heterologous expression at Novozymes A/S using *Aspergillus oryzae* as the expression host, essentially as discussed in Biely *et al.* (2014 ▸). A novel band of about 35 kDa was observed in cultures of transformants that was not observed in cultures of the untransformed production strain. The expression level was investigated using SDS–PAGE for several transformants that appeared to express the recombinant arabinofuranosidase. After expression of the transformant with the highest expression level in a 1 l bioreactor, the culture broth was sterile-filtered to remove the mycelia. The filtrated broth was brought to 1.8 *M* ammonium sulfate, and after filtration (0.22 µm PES filter; Nalge Nunc International, Nalgene labware catalogue No. 595-4520) the filtrate was loaded onto a Phenyl Sepharose 6 Fast Flow column (high sub; GE Healthcare, Piscataway, New Jersey, USA) equilibrated with 25 m*M* HEPES pH 7.0 with 1.8 *M* ammonium sulfate; the column was washed with three column volumes of 25 m*M* HEPES pH 7.0, 1.0 *M* ammonium sulfate and bound proteins were eluted with 25 m*M* HEPES pH 7.0. The fractions were pooled and applied onto a Sephadex G-25 column (GE Healthcare) equilibrated with 25 m*M* HEPES pH 7.5. The fractions were applied onto a SOURCE 15Q column (GE Healthcare) equilibrated with 25 m*M* HEPES pH 7.5 and the bound proteins were eluted with a linear gradient from 0 to 1000 m*M* sodium chloride over ten column volumes. Fractions were analyzed by SDS–PAGE and those containing the arabinofuranosidase were combined.

The synthesis of AraDNJ was carried out using literature procedures (Jones *et al.*, 1985 ▸; Naleway *et al.*, 1988 ▸).

### 2.2. Crystallization   

Crystallization screening was carried out by sitting-drop vapour diffusion with drops set up using a Mosquito Crystal liquid-handling robot (TTP Labtech, England) with 150 nl protein solution plus 150 nl reservoir solution in 96-well format plates (MRC 2-well crystallization microplates, Swissci, Switzerland) equilibrated against 54 µl reservoir solution. Experiments were carried out at room temperature using several commercial screens.

Extensive screening was carried out with no promising hits. As a final resort, the sample was subjected to shallow-gradient ion exchange in Tris–HCl pH 8.5. The resultant peak was asymmetric and the conditions of the run were adjusted to optimize the separation of different regions of the peak (whole gradient 0–1 *M* NaCl, peak separation at 10–20% of elution buffer). Fractions for these regions were pooled separately and concentrated. Crystallization was set up with protein fractions from the beginning of the peak. Crystallizations were performed both with and without the inhibitor AraDNJ which, when used, was mixed with the protein to give a final concentration of 5 m*M*. The best hit was obtained for protein in complex with the inhibitor from Crystal Screen HT condition G3 (0.01 *M* zinc sulfate, 0.1 *M* MES pH 6.5, 25% PEG 550 MME); this was chosen to make a seeding stock for further optimizations.

The seeding stock was prepared and microseed matrix screening (MMS; for a recent review, see D’Arcy *et al.*, 2014 ▸) was carried out using an Oryx robot (Douglas Instruments) according to the published protocols (Shaw Stewart *et al.*, 2011 ▸; Shah *et al.*, 2005 ▸) with two screens, Crystal Screen HT and JCSG, as well as a number of optimizations of the hit conditions. Diffraction-quality crystals were obtained from JCSG screen conditions B2, G7 and G10. That used for data collection was obtained from condition G10, *i.e.* 30% PEG 2K MME, 0.2 *M* KBr. The crystals were cryoprotected by adding PEG 3350 to the mother liquor in a 1:2 ratio (3 µl PEG + 6 µl mother liquor), which corresponded to 16.6% PEG 3350 and 20% PEG 2K in the final cryoprotectant solution. Crystallization conditions are shown in Table 1 ▸.

### 2.3. Data collection and processing   

All computations were carried out using programs from the *CCP*4 suite (Winn *et al.*, 2011 ▸) unless otherwise stated. The data were collected on beamline I04-1 at Diamond Light Source (DLS) to 1.2 Å resolution and were processed with *xia*2 (Winter *et al.*, 2013 ▸). Data-collection and processing statistics are given in Table 2 ▸.

### 2.4. Structure solution and refinement   

The structure was solved by *MOLREP* (Vagin & Teplyakov, 2010 ▸) using *S. coelicolor* α-l-arabinofuranosidase (PDB entry 3wmy; Maehara *et al.*, 2014 ▸) as the search model. Chain tracing used *Buccaneer*, and the structure was refined with *REFMAC* (Murshudov *et al.*, 2011 ▸) iterated with manual model correction using *Coot* (Emsley *et al.*, 2010 ▸). The quality of the final model was validated using *MolProbity* (Chen *et al.*, 2010 ▸) as part of the *PHENIX* package (Adams *et al.*, 2011 ▸). The final refinement statistics are given in Table 3 ▸. The structure has been deposited in the PDB as entry 6f1j.

### 2.5. Isothermal titration calorimetry   

Ligand affinity was measured using isothermal titration calorimetry (ITC). ITC was performed at 25°C in 25 m*M* HEPES pH 7.0, 100 m*M* NaCl using a Malvern MacroCal Auto-iTC200 calorimeter. The ligand in the syringe was at 1.8 m*M* and was titrated into a cell containing a 112 µ*M* solution of the enzyme. Assays were performed in duplicate. The dissociation constant was calculated using the *PEAQ-ITC Analysis* software (Malvern).

## 3. Results and discussion   

**Figure 2:**
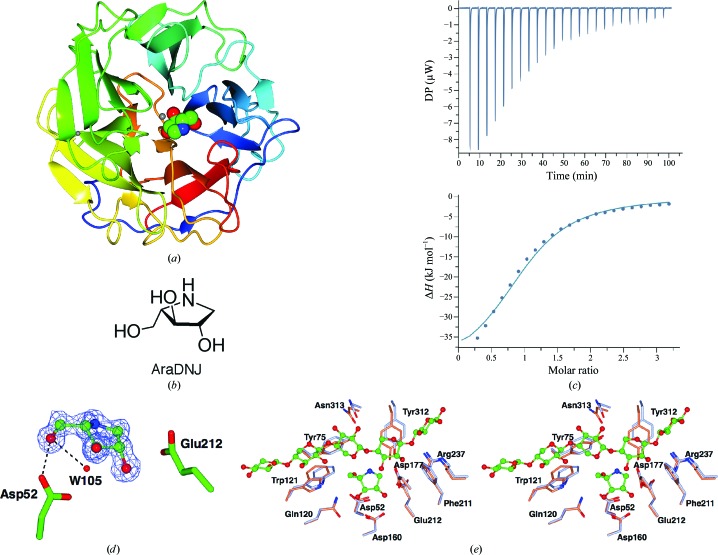
Three-dimensional structure and ligand binding of the *T. pinophilus* GH62 arabinofuranosidase in complex with the inhibitor AraDNJ. (*a*) Three-dimensional structure colour-ramped from the N-terminus (blue) to the C-terminus (red). Metal ions are shown as shaded spheres and AraDNJ as a CPK model. (*b*) The chemical structure of AraDNJ. (*c*) ITC data for AraDNJ binding (*K*_d_ of 24 ± 0.4 µ*M*). (*d*) Observed electron density for AraDNJ bound to GH62, 2*F*_o_ − *F*_c_ (maximum-likelihood/σ_A_-weighted) at 1.25 Å contoured at 1σ. The catalytic acid Glu212 and base Asp52 are shown, along with a water molecule poised for nucleophilic attack. (*e*) Partial overlay of the *T. pinophilus* GH62 arabinofuranosidase (brown with AraDNJ in green) with the *S. coelicolor* GH62 arabinofuranosidase (PDB entry 3wn2; pale blue with xylopentaose in green), highlighting the highly conserved binding centre and the recognition apparatus for the arabinoxylan chain. Structural figures were drawn with *CCP*4*mg* (McNicholas *et al.*, 2011 ▸).

The structure (PDB entry 6f1j) was solved and refined at 1.25 Å resolution (Table 3 ▸). The protein chain can be traced from residues 25 through to 325 and contains both structural calcium and zinc ions. The five-bladed β-propeller structure (Fig. 2 ▸ *a*) bears a strong similarity to those of previously published GH62 enzymes, notably those from *S. coelicolor* (Maehara *et al.*, 2014 ▸) and *S. thermoviolaceus* (Wang *et al.*, 2014 ▸); 300 residues align with 72 and 69% sequence identity and r.m.s. C^α^ deviations of 0.58 and 0.68 Å, respectively, as reflected by high *PDBeFold* (Krissinel & Henrick, 2004 ▸) *Q* scores of 0.95 and 0.94, respectively. There are two subunits in the asymmetric unit with high structural similarity (r.m.s.d. of 0.22 Å), with some conformational differences on the outer surfaces, in particular in the region of crystal contacts.

Of the two metal ions, the Ca^2+^ ion is located essentially as reported previously, for example in the *S. coelicolor* enzyme (Maehara *et al.*, 2014 ▸). However, this structural Ca^2+^ ion (which is close to, but does not impinge on, the active centre) is coordinated by six water molecules and a carboxylate O atom from Glu215. This is different to previous structures, in which the Ca^2+^ ion was coordinated by a His and Gln pair, which are replaced here by a water molecule hydrogen-bonded to Ser278 (in place of the His) and directly to Glu215 (in place of the Gln observed previously). In the *T. pinophilus* enzyme there are additional Zn^2+^ ions derived from the ‘seeding stock’ (see above) element of the crystallization conditions. One of those bridges the *A* and *B* molecules in the lattice, presumably aiding lattice formation, with coordination from His180 from molecule *A* and the amino-terminal NH_2_ and carbonyl groups of Ser24 and the side chain of Glu220 from molecule *B*. Another Zn^2+^ ion is coordinated by Glu88 from molecule *B*, His180 from the symmetry-related molecule *B* and three waters.

The structure of the *T. pinophilus* GH62 arabino­furanosidase was determined in the presence of the putative arabinofuranosidase inhibitor AraDNJ (Fig. 2 ▸ *b*), which allows further confirmation of the catalytic apparatus. This compound has found use in studies of other arabino­furanosidases (Axamawaty *et al.*, 1990 ▸; Hemsworth *et al.*, 2016 ▸) as well as as a scaffold for developing inhibitors of other glycosidases (Siguier *et al.*, 2014 ▸; Mena-Barragán *et al.*, 2016 ▸). Azasugars and iminosugars are generally considered to be good inhibitors of retaining glycoside hydrolases by virtue of their endocyclic N atom, which can be protonated, thus mimicking the putative positive charge that is thought to exist in the transition state(s) during glycoside hydrolysis. In addition, the N atom provides adventitious interactions with both the acid/base and the nucleophile in the active sites of these enzymes (see, for example, Gloster *et al.*, 2007 ▸). GH62 enzymes are inverting and thus do not have a suitably positioned nucleophile. It was therefore surprising to us that AraDNJ acted as an inhibitor with well resolved density. The binding constant for AraDNJ was therefore determined by isothermal titration calorimetry (Fig. 2 ▸ *c*), revealing a surprisingly tight *K*_d_ of 24 ± 0.4 µ*M*. It is rare in glycosidases that iminosugars bind so well to the glycosidase active site without a close enzyme-derived nucleophilic interaction, but other examples include CAZY family GH6, where cellobio-derived isofagomines have been used to good effect, even reporting on the substrate distortions involved in catalysis (Gloster *et al.*, 2007 ▸). Here, AraDNJ binds in a potentially transition-state-mimicking ^4^ *E* conformation. As might be expected, AraDNJ binds in the same location as observed for Araf itself (see, for example, PDB entry 4o8o; Wang *et al.*, 2014 ▸), making similar hydrogen bonds from O2 and O3 to Asp160, from O3 to Gln120 and from O56 to Asp52. There is also a potential hydrophobic contact with the side chain of Ile159. There is no direct interaction of the positively charged N atom (here replacing the endocyclic O atom of arabinose), but the structure reveals a water molecule poised 3.1 Å ‘below’ the furanose ring, where it hydrogen-bonds to Asp52, the putative catalytic base, consistent with previous studies (Maehara *et al.*, 2014 ▸; Wang *et al.*, 2014 ▸) and the inverting mechanism (Fig. 1 ▸ *b*). Glu212, the putative acid, is placed for lateral *anti* protonation of any departing group (Fig. 2 ▸ *d*). Notably, the positively charged N atom lies exactly where the positively charged N atom of published Tris complexes of homologues sits (see, for example, PDB entry 3wn2, the *S. coelicolor* GH62 enzyme; Maehara *et al.*, 2014 ▸), highlighting that these enzymes have evolved to stabilize the positively charged transition state, even without the aid of the direct charge–charge interactions available to retaining enzymes.

The *T. pinophilus* GH62 enzyme in complex with AraDNJ, viewed in light of past work on xylooligosaccharide complexes of GH62 enzymes, provides further insight into the mechanisms by which GH62 enzymes remove the arabinofuranoside decorations from arabinoxylan. An overlay with the xylopentaose complex (PDB entry 3wn2) of the *S. coelicolor* GH62 enzyme (Maehara *et al.*, 2014 ▸; Fig. 2 ▸ *e*) shows how the interacting surface for the xylan chain is highly conserved between the two enzymes, with both aromatic platforms (Phe211, Tyr312 and Trp121) and some hydrogen-bonding interactions (Arg237, Asn313 and Asp177) being invariant, suggesting that ligand recognition is similar. Indeed, C1 of the AraDNJ complex lies 1.9 Å from the O3 atom of the ‘second’ (from the reducing end) xylose moiety in PDB entry 3wn2, highlighting how the *T. pinophilus* GH62 enzyme could act as an arabinofuranosidase that is active on O3-substituted xylans, as was proposed originally for the *S. coelicolor* GH62 enzyme (Maehara *et al.*, 2014 ▸), although it is possible to also consider action at the O2 position should the xylan chain occasionally be reversed through the active site (which is possible with xylans given their internal pseudo-symmetry).

The *T. pinophilus* GH62 enzyme thus adds to the growing literature surrounding these key players in natural and industrial arabinoxylan degradation. It demonstrates how arabinofuranoside mimics lie in the active site of the enzyme and how the enzyme recognizes and cleaves arabinoxylan. Furthermore, the nonclassical application of an iminosugar-based glycosidase inhibitor to study inverting-enzyme structure and function should encourage the further non-intuitive application of such compounds in the future.

## Supplementary Material

PDB reference: *Talaromyces pinophilus* arabinofuranosidase, complex with AraDNJ, 6f1j

## Figures and Tables

**Table 1:** Crystallization

Method	Vapour diffusion, sitting drop; MMS
Plate type	MRC 2-well crystallization microplate, Swissci, Switzerland
Temperature (K)	293
Protein concentration (mg ml^−1^)	25
Buffer composition of protein solution	20 m*M* Tris–HCl pH 8.5, 150 m*M* NaCl
Composition of reservoir solution	30% PEG 2K MME, 0.2 *M* KBr
Volume and ratio of drop	300 nl total, 1:1 ratio
Volume of reservoir (µl)	54

**Table 2:** Data-collection statistics Values in parentheses are for the outer shell.

Diffraction source	I04-1, DLS
Wavelength (Å)	0.93
Temperature (K)	100
Detector	PILATUS 6M-F
Crystal-to-detector distance (mm)	254.2
Rotation range per image (°)	0.1
Total rotation range (°)	180
Exposure time per image (s)	0.0375
Space group	*P*2_1_
*a*, *b*, *c* (Å)	43.83, 88.97, 72.66
α, β, γ (°)	90, 95.22, 90
Mosaicity (°)	0.11
Resolution range (Å)	33.52–1.25 (1.27–1.25)
Total No. of reflections	457639 (14559)
No. of unique reflections	149344 (6813)
Completeness (%)	98 (91)
CC_1/2_ †	0.998 (0.79)
Multiplicity	3.1 (2.1)
〈*I*/σ(*I*)〉	13.1 (2.9)
*R*_merge_	0.044 (0.28)
*R*_r.i.m._ ‡	0.052 (0.34)
Overall *B* factor from Wilson plot (Å^2^)	5.1

† CC_1/2_ values for *I*_mean_ are calculated by splitting the data randomly into two half data sets.

‡ Estimated *R*_r.i.m._ = *R*_merge_[*N*/(*N* − 1)]^1/2^, where *N* is the data multiplicity, and *R*_merge_ is defined as 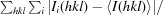 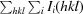, where *I*(*hkl*) is the intensity of the reflection.

**Table 3:** Structure solution and refinement

Resolution range (Å)	33.52–1.25
Completeness (%)	97.8
No. of reflections	
Working set	141792
Test set	7088
Final *R*_cryst_	0.120
Final *R*_free_	0.136
Cruickshank DPI	0.037
No. of subunits in the asymmetric unit	2
R.m.s. C^α^ deviation between subunits (Å)	0.221
No. of non-H atoms	
Protein	4698
Ion	4
Ligand	18
Water	658
Total	5378
R.m.s. deviations	
Bonds (Å)	0.014 (0.020)
Angles (°)	1.5 (1.9)
Average *B* factors (Å^2^)	
Protein	
Chain *A*	7.3
Chain *B*	7.7
Ions	
Ca^2+^	3.3
Zn^2+^ (1st)	8.8
Zn^2+^ (2nd)	8.4
Ligand	6.7
Water	18.8
Ramachandran plot†	
Favoured (%)	96.4
Outliers (%)	0.33
*MolProbity* score	0.85

† Ramachandran plot analysis was carried out by *MolProbity* (Chen *et al.*, 2010 ▸).
